# Role of Cyclic Nucleotide-Gated Channels in the Modulation of Mouse Hippocampal Neurogenesis

**DOI:** 10.1371/journal.pone.0073246

**Published:** 2013-08-22

**Authors:** Maria Vittoria Podda, Roberto Piacentini, Saviana Antonella Barbati, Alessia Mastrodonato, Daniela Puzzo, Marcello D’Ascenzo, Lucia Leone, Claudio Grassi

**Affiliations:** 1 Institute of Human Physiology, Medical School, Università Cattolica, Rome, Italy; 2 Section of Physiology, Department of Bio-Medical Sciences, University of Catania, Catania, Italy; University of Houston, United States of America

## Abstract

Neural stem cells generate neurons in the hippocampal dentate gyrus in mammals, including humans, throughout adulthood. Adult hippocampal neurogenesis has been the focus of many studies due to its relevance in processes such as learning and memory and its documented impairment in some neurodegenerative diseases. However, we are still far from having a complete picture of the mechanism regulating this process. Our study focused on the possible role of cyclic nucleotide-gated (CNG) channels. These voltage-independent channels activated by cyclic nucleotides, first described in retinal and olfactory receptors, have been receiving increasing attention for their involvement in several brain functions. Here we show that the rod-type, CNGA1, and olfactory-type, CNGA2, subunits are expressed in hippocampal neural stem cells in culture and *in situ* in the hippocampal neurogenic niche of adult mice. Pharmacological blockade of CNG channels did not affect cultured neural stem cell proliferation but reduced their differentiation towards the neuronal phenotype. The membrane permeant cGMP analogue, 8-Br-cGMP, enhanced neural stem cell differentiation to neurons and this effect was prevented by CNG channel blockade. In addition, patch-clamp recording from neuron-like differentiating neural stem cells revealed cGMP-activated currents attributable to ion flow through CNG channels. The current work provides novel insights into the role of CNG channels in promoting hippocampal neurogenesis, which may prove to be relevant for stem cell-based treatment of cognitive impairment and brain damage.

## Introduction

In the adult mammalian brain, new neurons are continuously generated in two regions: the subgranular zone (SGZ) of the hippocampal dentate gyrus (DG) and the subventricular zone (SVZ) of the lateral ventricles [1–3]. A so called “neurogenic niche” regulates the sequential steps of adult SGZ and SVZ neurogenesis ensuring continuous neuronal production while maintaining the neural stem cell (NSC) pool [4]. Adult neurogenesis in the hippocampus contributes to learning and memory [5–7], and increasing evidences have revealed that its alteration is associated with neurodegenerative and neuropsychiatric diseases, including Huntington’s disease [8], Parkinson’s disease [9], Alzheimer’s disease [10], schizophrenia and depression [11–15].

Many studies have demonstrated that several extrinsic factors modulate adult hippocampal neurogenesis including a broad range of physiological, environmental, physical and pharmacological stimuli [16–20]. Although decades have elapsed between the initial discovery of postnatal mammalian neurogenesis and *in vitro* derivation of multipotent NSCs from the adult mouse brain, fundamental information is still lacking on the intrinsic regulatory mechanisms controlling proliferation, differentiation and NSC survival. Understanding molecular mechanisms controlling adult neurogenesis is of great value given its role in the physiology of brain function as well as in the pathophysiology of neurodegenerative and neuropsychiatric diseases.

There is evidence indicating that neurogenesis is positively affected by cGMP and cGMP sparing agents such as sildenafil or tadalafil [21–26]. Some of the cGMP effects have been linked to activation of intracellular pathways by nitric oxide (NO) [27], which is a well recognized mediator involved in the regulation of adult neurogenesis both in basal condition and following brain injury [28–31]. However, the molecular mechanisms underlying the effects of cGMP on basal neurogenesis have not been well characterized. Within this scenario, we investigated the role of a class of ion channels directly gated by cyclic nucleotides, namely, the cyclic nucleotide-gated (CNG) channels, in the modulation of adult hippocampal neurogenesis*.*

CNG channels are heteromeric complexes made up of principal and modulatory subunits. In mammals, four A subunits and two B subunits have been described. The rod-type CNGA1 subunit, the olfactory-type CNGA2 subunit, and the cone-type CNGA3 subunit each form a functional channel on their own, the properties of which can be modified by heteromerization with the A4 subunit or with either of the two β subunits (CNGB1 and CNGB3). CNGA1 and CNGA3 channels are activated primarily by cGMP, whereas the CNGA2 channel is equally sensitive to physiological concentrations of cAMP and cGMP [32,33]. An increasing body of evidence indicates that these voltage-independent cation channels, firstly described as key elements in signal transduction mechanism in the retina and olfactory epithelium [32,34,35], are expressed in the CNS by neuronal and glial cells and influence a variety of cellular processes and functions including neuronal excitability, neurotransmitter release, synaptic plasticity, growth cone guidance and synaptic bouton maturation [36–43].

Here we show that the rod-type and olfactory-type CNGA subunits are expressed in both cultured hippocampal NSCs and the neurogenic niche of the mouse hippocampus *in situ*. In cultured NSCs electrophysiological studies revealed cGMP-activated currents with pharmacological and biophysical features suggestive of ion flow through CNG channels. The pharmacological blockade of CNG channels reduced NSC differentiation toward the neuronal phenotype. Enhancement of neuronal differentiation by exogenous cGMP was also prevented by CNG channel blockade.

## Materials and Methods

### Ethics Statement

All animal procedures were approved by the Ethics Committee of the Catholic University and were fully compliant with Italian (Ministry of Health guidelines, Legislative Decree No. 116/1992) and European Union (Directive No. 86/609/EEC) legislation on animal research. Efforts were made to limit the number of animals used and to minimize their suffering.

### Hippocampal neural stem cell culture

**Figure 1:**
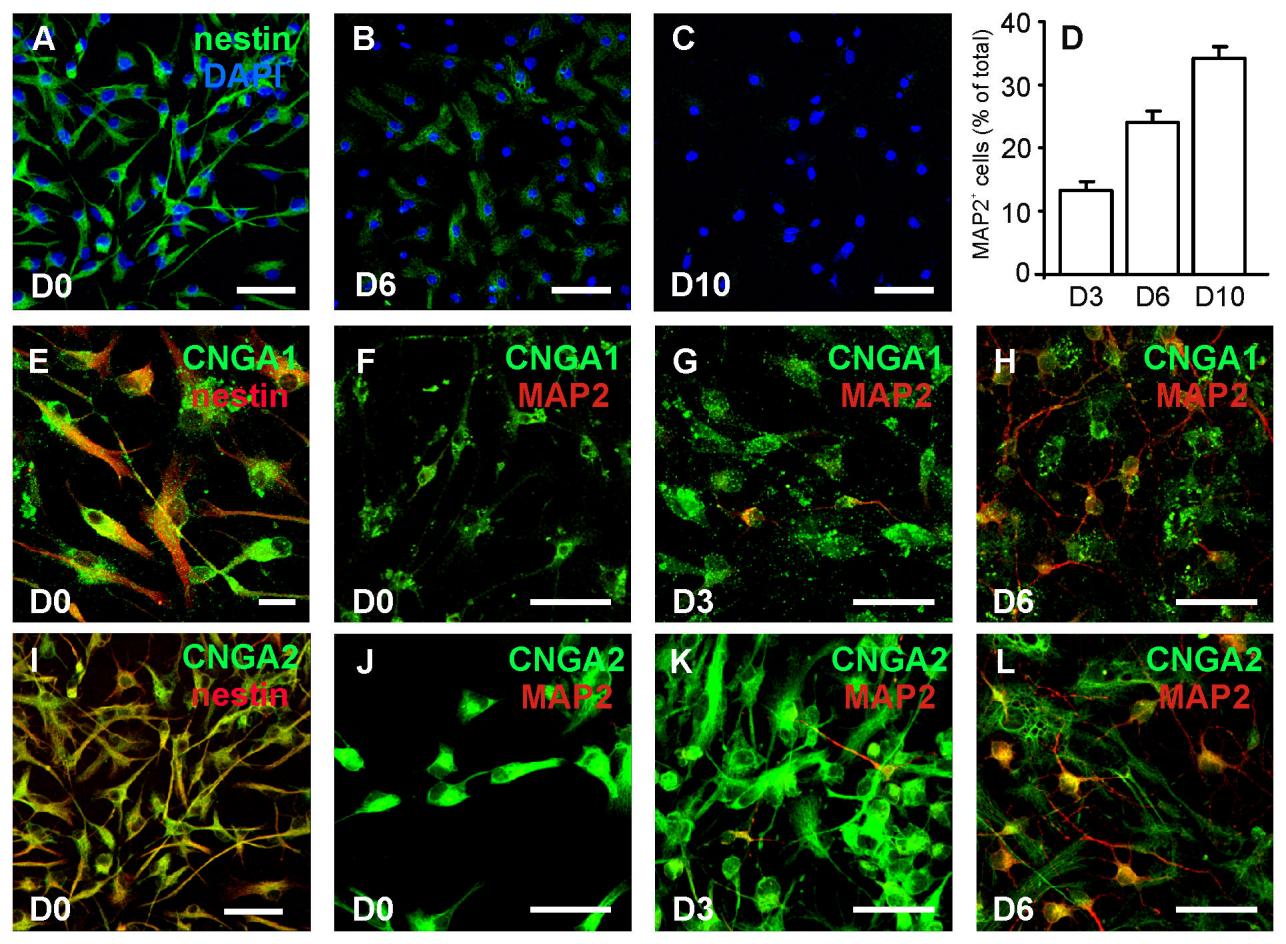
Cultured hippocampal NSCs express CNG channels. (**A**–**D**) Time course of hippocampal NSC differentiation assessed by immunofluorescence. The vast majority of neurosphere-derived cells grown in the proliferation medium (D0) were positive for nestin (**A**); during differentiation this immunoreactivity diminished (**B**) and ultimately disappeared on D10 (**C**). Cell nuclei (DAPI^+^) are labeled in blue. The percentages of cells identified as neurons by MAP2 immunoreactivity significantly increased during the 10 days of differentiation (**D**). Undifferentiated NSCs, characterized by positivity for nestin and lack of MAP2 immunoreactivity, showed both CNGA1 (**E**,**F**) and CNGA2 staining (**I**,**J**). NSCs differentiating toward neuronal phenotype (MAP2^+^) exhibited CNGA1 (**G**,**H**) and CNGA2 (**K**,**L**) labeling. Scale bars: 50 µm.

Adult hippocampal NSC culture were isolated according to previously published protocols [44]. Briefly, brains of newborn (0-1 day old) C57bl/6 mice were microdissected to obtain the hippocampal region upon sagittal sectioning. Tissues were finely minced and digested by accutase (in DPBS, 0.5 mM EDTA; Innovative Cell Tecnologies, Inc., San Diego, CA, USA) at 37°C for 30 min. After centrifugation, cells were carefully dissociated by passaging in fire-polished Pasteur pipettes and resuspended in NeurobsalA medium, supplemented by 2% B27 (Gibco, Grand Island, NY, USA), Glutamax (0.5 mM; Invitrogen, Carlsbad, CA), mouse fibroblast growth factor 2 (FGF2, 10 ng/ml; Invitrogen), epidermal growth factor (EGF, 10 ng/ml; Invitrogen), mouse platelet-derived growth factor bb (PDGFbb, 10 ng/ml; Invitrogen). Cells were seeded onto 25-cm^2^ T-flask and incubated at 37°C in 5% CO_2_ atmosphere. During the first week NSCs began to form neurospheres *in vitro*. At 2-day intervals, the neurospheres were collected, and passaged by a gently enzymatic and mechanical dissociation. These processed NSCs retained the potential to grow infinitely, as demonstrated by the percentage of the cells positive for nestin, a marker for immature neural progenitors (Figure 1A).

To obtain monolayer cultures, neurospheres of established cultures were passaged by enzymatic and mechanical dissociation and plated as single cells onto Matrigel Matrix (Becton Dickinson, Franklin Lakes, NJ) pre-coated Petri dishes. NSCs cultured in the medium described above, thereinafter referred to as “proliferation medium”, remained in an undifferentiated state and proliferated. To induce differentiation, NSCs were cultured for up to 10 days in a medium defined as “differentiation medium”, in which the FGF-2, EGF and PDGFbb had been replaced with 1% FBS.

### Proliferation analysis by BrdU incorporation

NSC proliferation was studied by 5-bromo-2’-deoxyuridine (BrdU, Sigma, Milan, Italy) incorporation. Following BrdU application (5 µM) to the NSC culture medium for 4 h, BrdU^+^ cells were immunocytochemically identified to quantify cell replication during exposure to the thymidine analogue.

### NSC culture drug treatment

8-bromoguanosine 3’, 5’-cyclic monophosphate sodium salt (8-Br-cGMP) and KT5823 were from Sigma. l-(-)-*cis*-diltiazem hydrochloride (LCD) was from Enzo Life Sciences. Stock solutions were made up in distilled water except for KT5823 which was dissolved in DMSO.

The different drugs were added to proliferation medium for 24 h and then tested for BrU incorporation. Drugs were added to proliferation medium for three days (D1 to D3) then diluted when half-medium changes were performed at D3 and D6.

### Immunocytochemistry

NSCs were processed for immunocytochemistry, as previously described [45]. Briefly, cells were fixed with 4% paraformaldehyde in PBS for 10 min at room temperature (RT); rinsed twice in PBS; and permeabilized with PBS/TritonX-100 (0.3%) for 15 min. For BrdU detection assays only, permeabilization was followed by 30 min incubation in 2N HCl for DNA denaturation. Cells were then blocked with 0.3% bovine serum albumin (BSA) in PBS and incubated overnight (at +4°C) with one of the following antibodies (diluted in PBS/BSA): rabbit anti-glial fibrillary acid protein (GFAP) for astroglia (1:500, Chemicon International, Inc., Temecula, CA); mouse anti-MAP2 (1:300, clone HM-2; Sigma) for neurons; rabbit anti-CNGA1 (1:800, Immunological Sciences, Rome, Italy) or rabbit anti-CNGA2 (1:100, Alomone Laboratories, Jerusalem, Israel); mouse anti-BrdU (1:200, clone 85-2C8, Ylem, Italy or 1:300, clone BMC9318, Chemicon). The following day, cells were washed and incubated for 90 min at RT with secondary antibodies diluted in PBS: goat anti-rabbit Alexa Fluor 488 (1:1,000; Invitrogen) and rhodamine-conjugated goat anti-mouse (1:300; Millipore, Billerica, MA). Cell nuclei were counterstained with 4'6-diamidino-2-phenylindole (DAPI; 0.5 µg/ml; Invitrogen), and the cells were coverslipped with ProLong Gold antifade reagent (Invitrogen).

Images were obtained with a confocal laser scanning system (TCS-SP2, Leica Microsystems, GmbH, Wetzlar, Germany) equipped with an Ar/ArKr laser (for 488-nm excitation) and a HeNe laser (for 543-nm excitation). DAPI staining was imaged by two-photon excitation (740 nm, < 140 fs, 90 MHz) performed with an ultrafast, tunable, mode-locked Ti: Sapphire laser (Chameleon, Coherent Inc., Santa Clara, CA).

Double-blind counts of immunoreactive cells were performed in at least 10 random 40× magnification fields for each experiment, and data were expressed as percentages of the total number of cells within the same fields (reflected by DAPI-counterstained nuclei). Experiments were repeated 4 to 7 independent times in duplicate.

To determine specificity of CNGA1 and CNGA2 staining, each antibody was pre-incubated with five-fold (by weight) of an equal amount of its specific peptide for 2 h at RT and applied in place of the primary antibody, according to the manufacturer’s data sheets. Controls for immunofluorescence were obtained by omitting the primary antibody or substituting the primary antibody with the host IgG from which the antibody was generated. In all cases no artifactual labeling was ever detected.

### Western Immunoblot analysis

For extraction of total proteins, pelleted NSCs were placed in ice-cold RIPA buffer (Pierce, Rockford, IL, USA) containing 50 mM Tris, 150 mM NaCl, 1 mM EDTA, 1% sodium deoxycholate, 1% Triton X-100, 0.1% sodium dodecyl sulfate (SDS), and 1× protease, phosphatase-1, and phosphatase-2 inhibitor cocktails (Sigma). The lysates were centrifuged (12,000 rpm, 20 min, 4°C), and a 5-μl aliquot of the supernatant was assayed to determine the protein concentration (microBCA kit, Pierce). SDS-PAGE reducing sample buffer was added to the supernatant, and samples were heated to 95°C for 5 min. Protein lysates (50–100 μg) were loaded onto 8% Tris-glycine polyacrylamide gels for electrophoretic separation. Colorburst^TM^ Electrophoresis markers (Sigma) were used as molecular mass standards. Proteins were then transferred onto nitrocellulose membranes (37 V overnight at 4°C in transfer buffer containing 25 mM Tris, 192 mM glycine, 0.1% SDS, and 20% methanol). After staining with Ponceau S (Sigma), the membranes were incubated for 1 h with blocking buffer (5% skim milk in Tris-buffered saline containing 0.5% Tween 20, TBST) and then overnight at 4°C with primary antibodies against CNGA1 or CNGA2 (described above), both diluted 1:200, or antibody against actin (1:1,000; Sigma).

The antibody specificity was verified by peptide blockade performed by incubating the primary antibody with fivefold excess of the specific immunizing peptide, for 2 h at RT and then diluting the antibody-peptide solution as described for the primary antibody [46]. These experiments were performed on whole cell lysates of NSCs (D3) and of mouse hippocampal tissue (used as positive control).

After three 10-min rinses in TBST, membranes were incubated for 1 h at RT with horseradish peroxidase (HRP)-conjugated anti-rabbit or anti-mouse antibodies (1:2,500; Cell Signaling Technology Inc., Danvers MA). The membranes were then washed and the bands visualized with the aid of an enhanced chemiluminescence detection kit (GE Healthcare, Buckinghamshire, UK).

### Immunohistochemistry

Three 28-day-old male C57bl/6 mice were anesthetized with a cocktail of ketamine (100 mg/ml) and medetomidine (1 mg/ml) and transcardially perfused with Ringer’s solution followed by 4% paraformaldehyde fixative solution. Their brains were removed, post-fixed overnight at 4°C, and transferred to a solution of 30% sucrose in PBS where they remained for 2 days. Coronal sections (40 µm thick) were then cut with a vibratome (VT1000S, Leica), floated in ice-cold PBS and then transferred to cryoprotectant and stored at -20°C [47].

Sections were blocked for 1 h at RT in 1% BSA solution containing 10% goat serum and 0.5% Triton X-100 (Sigma) and incubated for 48 h at 4^°^C with rabbit polyclonal anti-CNGA2 (1:60; Alomone) or anti-CNGA1 (1:200, Immunological Sciences). The slices were then washed several times in PBS and reincubated with the secondary antibody, donkey anti-rabbit Alexa-488 (1:500; Invitrogen). For double labeling experiments cells were incubated for 2 h with one of the following: mouse monoclonal anti-nestin antibody (1:100; Millipore); rabbit polyclonal anti-DCX antibody (1:200; Cell Signaling). These markers were then visualized with Alexa-546 goat anti-mouse secondary antibody (1:500; Invitrogen) and Alexa-488 donkey anti-rabbit antibody labeling (1:500; Invitrogen). Finally, slices were incubated with DAPI (0.5 µg/ml; Invitrogen) to stain the cell nuclei, and the sections were mounted on glass slides and cover-slipped with ProLong Gold antifade reagent (Invitrogen). Pre-absorption and control experiments on all specimens were performed as described for immunocytochemistry. No specific signals were ever detected. Images were obtained as described for cell monolayers. Co-localization was quantified as Manders’ overlap coefficient (R: 0, no colocalization; 1, perfect colocalization) [48] by using ImageJ software (http://rsbweb.nih.gov/ij/).

### CNG A antibodies and control peptides

The following primary CNG A antibodies were used in the study: rabbit polyclonal anti-CNGA2 (APC-045, Alomone) raised against a peptide KQNH EDDYL SDGIN TPEP, corresponding to residues 643-660 in the C terminal cytoplasmic domain of rat/mouse CNGA2; rabbit polyclonal anti-CNGA1 (AB81642) raised against synthetic peptide from the N-terminal region of mouse CNGA1 channel (residues 100-150). Control peptides used in this study were: CNGA2 (APC-045, Alomone) and peptide for AB81642, Immunological Science, provided by the manufacturer).

### Patch-clamp recordings

Whole-cell patch-clamp recordings were performed on NSCs after three days in differentiation medium (D3) with an Axopatch 200B amplifier (Molecular Devices, Sunnyvale, CA). Stimulation and data acquisition were performed with a Digidata 1440A Series interface and pClamp 10 software (Molecular Device). Data were filtered at 1 kHz and digitized at 10 kHz. Data were not corrected for the liquid junction potential (-5.1 mV).

All recordings were made at RT. Patch pipettes were prepared from borosilicate glass capillary tubes (Warner Instruments, Hamden, CT) with a P-97 Flaming-Brown micropipette puller (Sutter Instruments, Novato, CA, USA) and filled with an internal solution containing (in mM): 145 K-gluconate, 2 MgCl_2_, 0.1 EGTA, 2 Na _2_ATP and 10 Hepes (pH 7.2 with KOH; 290 mOsm/l). The external Tyrode’s solution contained the following (in mM): 150 NaCl, 4 KCl, 2 CaCl_2_, 1 MgCl_2_, 10 glucose, and 10 Hepes (adjusted to pH 7.4 with NaOH). Some recordings were performed by using a Cs-based internal solution, which contained (in mM): 110 CsCl, 10 TEA-Cl, 2 MgCl_2_, 10 EGTA, 10 Hepes, and 4 Mg-ATP (pH adjusted to 7.2–7.4; 290-300 mOsm/l). Patch pipette solutions contained the selective PKG inhibitor, KT5823 (1 µM).

All recordings were carried out using extracellular solution containing 0.5 μM TTX, to block voltage-dependent Na^+^ channels, 5 µM nifedipine, to block L-type voltage-gated Ca^2+^ channels, 2 mM cesium chloride and 10 mM TEA, to block voltage-dependent and leakage K^+^ channels [42,49]. External solutions containing test drugs were applied *via* a perfusion system consisting of a multibarrelled pipette placed within 100 µm of the patched cell and connected to syringes by means of Teflon tubes. The gravity-regulated flow rate (0.3–0.5 ml/min) allowed complete renewal of the extracellular environment in <1 second.

### Statistical analysis

Data are expressed as means ± SEM. Statistical significance was assessed with Student’s t test and Two-way ANOVA. The Dunnett’s post-hoc test was used for multiple comparisons. For experiments that included fewer than 8 observations, the Mann–Whitney test was used. The level of significance was set at 0.05.

## Results

### Neuronal differentiation of hippocampal NSCs

When hippocampal cells obtained from postnatal mice were cultured in the proliferation medium, differentiated neurons and glial cells died while NSCs proliferated and formed self-renewing, multipotent neurospheres. The neurosphere-forming NSCs retained their ability to differentiate into neurons, astrocytes, or oligodendrocytes in response to appropriate stimuli. The vast majority (>99%) of the cells obtained from mechanically and enzymatically dissociated neurospheres were immunoreactive for nestin (Figure 1A), a molecular marker of uncommitted neural progenitor cells. When cells were grown in the differentiation medium there were time-dependent reductions and ultimately disappearance of nestin positivity (Figure 1B,C) accompanied by progressive increases in immunoreactivity for the neuronal marker, MAP2. The percentages of cells expressing MAP2 rose from 13.3±1.4% at D3 (206/1640 cells) to 24.0±1.9% (576/2058) and 34.2±1.9% (1304/2898), at D6 and D10, respectively (Figure 1D). Neuronal differentiation and maturation of MAP2^+^ cells was characterized by complex arborizations of their processes and the development of mature neuron-specific currents, including those through voltage-gated sodium channels generating action potential and those through ionotropic glutamate receptors (data not shown).

Data were collected from undifferentiated cells at day 0 (D0) or from NSCs undergoing differentiation toward the neuronal phenotype. The D0 cells were studied 24 h after dissociation from neurospheres and plating in proliferation medium. The differentiating cells were studied after 3, 6, and 10 days’ culture in the differentiation medium.

### CNG channels are expressed in cultured hippocampal NSCs and in the neurogenic niche of the hippocampus *in situ*

To determine whether CNG channels have a role in hippocampal neurogenesis we first investigated their expression in cultured hippocampal NSCs either undifferentiated or differentiating towards the neuronal phenotype. We focused on CNGA1 and CNGA2 channel subtypes because their expression has been demonstrated in several brain areas [41,42,50,51]. We, therefore, double-labeled NSCs grown in proliferation medium with antibodies raised against either CNGA1 or CNGA2 and a marker of undifferentiated NSCs (i.e., nestin). Similarly, double labeling for CNG channels and the neuronal marker MAP2 was performed in differentiating NSCs.

Results showed that virtually all cells cultured in proliferation medium (nestin^+^, MAP2^-^) were immunostained with both CNGA1 (Figure 1E,F) and CNGA2 subunits (Figure 1I,J), thus indicating that both channels subtypes are expressed in undifferentiated cells. CNG channel immunoreactivities were also found in neuron differentiating NSCs at D3 (Figure 1G,K) and in more mature neurons at D6 (Figure 1H,L), as shown by co-labeling with the neuronal marker MAP2. At D6 strong CNGA2 immunoreactivity was also observed in cells with morphological features of astrocytes in accordance with our previously published observations [42].

**Figure 2:**
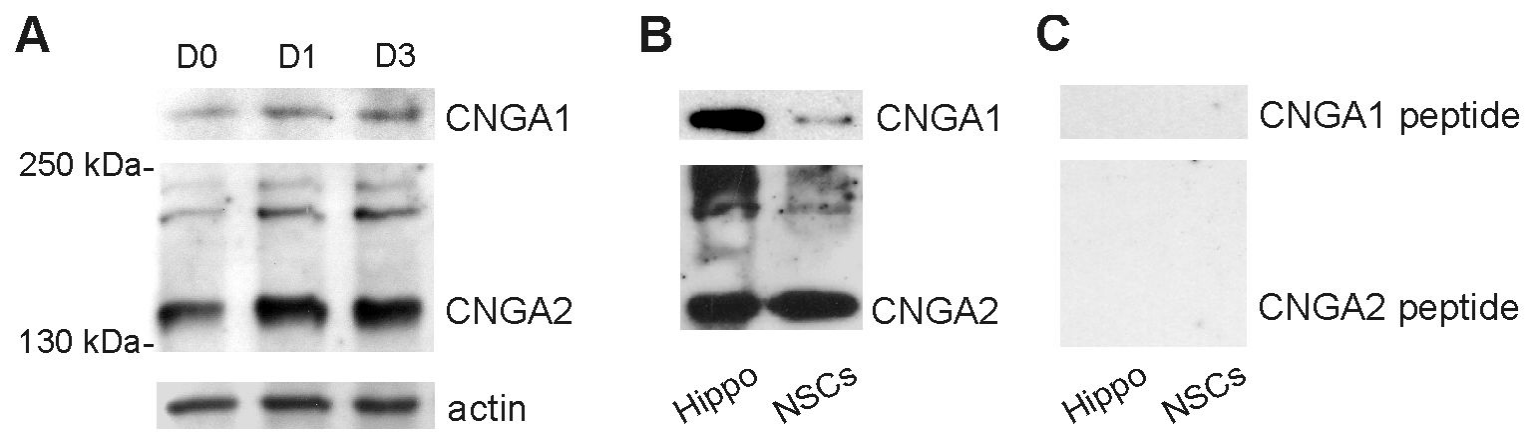
Western immunoblots showing **CNG A** protein expression in hippocampal NSCs. (**A**) Western immunoblots of whole-cell NSC lysates confirmed both CNGA1 and CNGA2 expression in undifferentiated (D0) and differentiating NSCs at D1 and D3. Loading control: actin. (**B**,**C**) Control experiments showing the specificity of CNG A immunoblotting. Bands corresponding to CNGA1 and CNGA2 proteins were detected in control samples (hippocampal tissue and NSCs at D3 (B) whereas no signals were detected when the antibodies were pre-absorbed with 5-fold excess of the appropriate control peptide (C).

Immunofluorescence data were confirmed by Western immunoblot analysis of total whole-cell lysates extracted from NSCs grown in proliferation (D0) and differentiation medium (D1 and D3) showing bands of expected size corresponding to CNGA1 and CNGA2 (Figure 2A) [42,46–49]. Additionally, immunoblots revealed higher levels of CNGA2 subunit expression compared to that of CNGA1 in undifferentiated as well as in differentiating NSCs. Pre-adsorption with immunizing peptides eliminated bands corresponding to CNGA1 and CNGA2 in whole cell lysates from NSCs (D3) and hippocampal tissue used as positive control [51,52], thus confirming the primary antibody specificity (Figure 2B,C).

**Figure 3:**
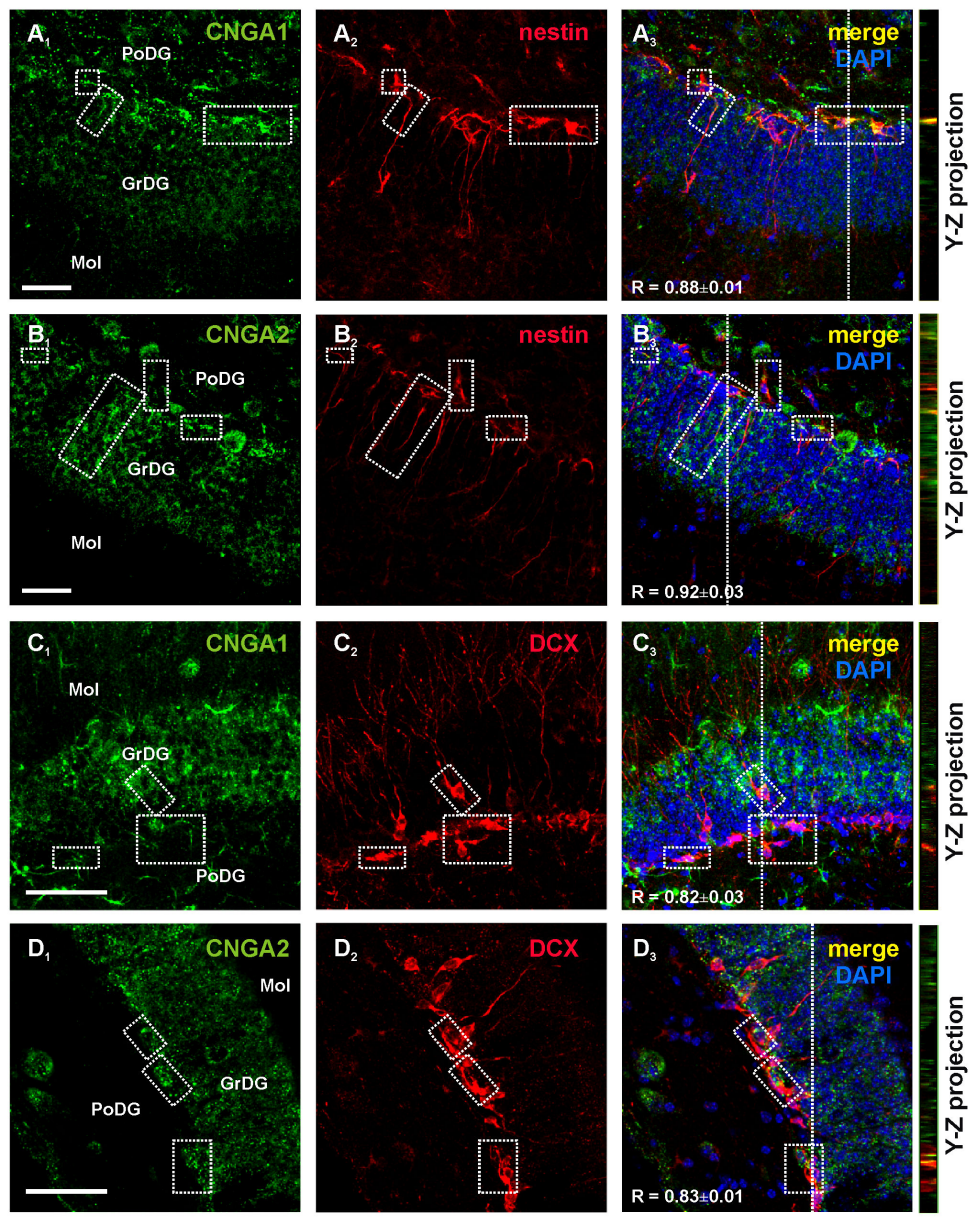
**CNG A** subunit expression in the hippocampal neurogenic region of the adult mouse brain. Confocal images of mouse hippocampal sections showed that CNGA1 and CNGA2 labelings (green, **A_1_** and **B_1_**, respectively) were present in nestin^+^ cells (red, **A_2_** and **B_2_**) in the subgranular zone of the DG. Panel **A_3_** shows merging of A_1_ and A_2_ and panel **B**_3_ shows merging of B_1_ and B_2_. Dotted boxes identify representative regions containing double labeled cells. CNGA1 and CNGA2 immunoreactivities (green, **C_1_** and **D_1_**) were also found in immature DCX^+^ neurons (red, **C_2_** and **D_2_**) in the DG. Panel **C_3_** and D_3_ show merging of C_1-2_ and of D_1-2_, respectively. Y-Z cross sections (referred to the dashed lines) from confocal Z-stack acquisitions show co-localization of CNGA channel subunits and nestin or DCX labeling. In panels A_3_-D_3_ R values represent mean Manders’ overlap coefficients (0, no colocalization; 1, perfect colocalization) calculated on boxed areas. GrDg: granular layer of the hippocampal DG; PoDG: polymorph layer of the DG; Mol: molecular layer of the DG.

We then evaluated CNG channel expression *in situ* in coronal sections of the hippocampus obtained from adult mice. Immunoreactivity for CNGA1 and CNGA2 was detected in the subgranular region of the DG and co-localized with nestin immunoreactivity in both soma and processes (Figure 3A and B, respectively). CNGA1^+^ and CNGA2^+^ cells were also found in the innermost cell layer of the DG and inside the granule cell layer. In these regions, CNGA subunit-positive cells co-labeled with the immature neuron marker, DCX (Figure 3C,D). Mature granule cells within the granule cell layer of the DG, identified by their location and morphology, were also positive to CNGA1 and CNGA2 immunolabelings (Figure 3A_1_-D_1_).

### CNG channel-like conductances in NSC-derived neurons

Our next step was to seek for electrophysiological evidence of CNG channel activation in differentiating NSCs. Patch-clamp recording were performed on D3 NSCs and they were restricted to cells with the morphological features that had been associated with the immunocytochemical marker of the neuronal phenotype. None of the recorded cells showed morphological phenotype and electrophysiological features (i.e., low input resistance and high resting potential) of astrocytes, that we previously demonstrated to express CNG channel-gated conductances [42].

**Figure 4:**
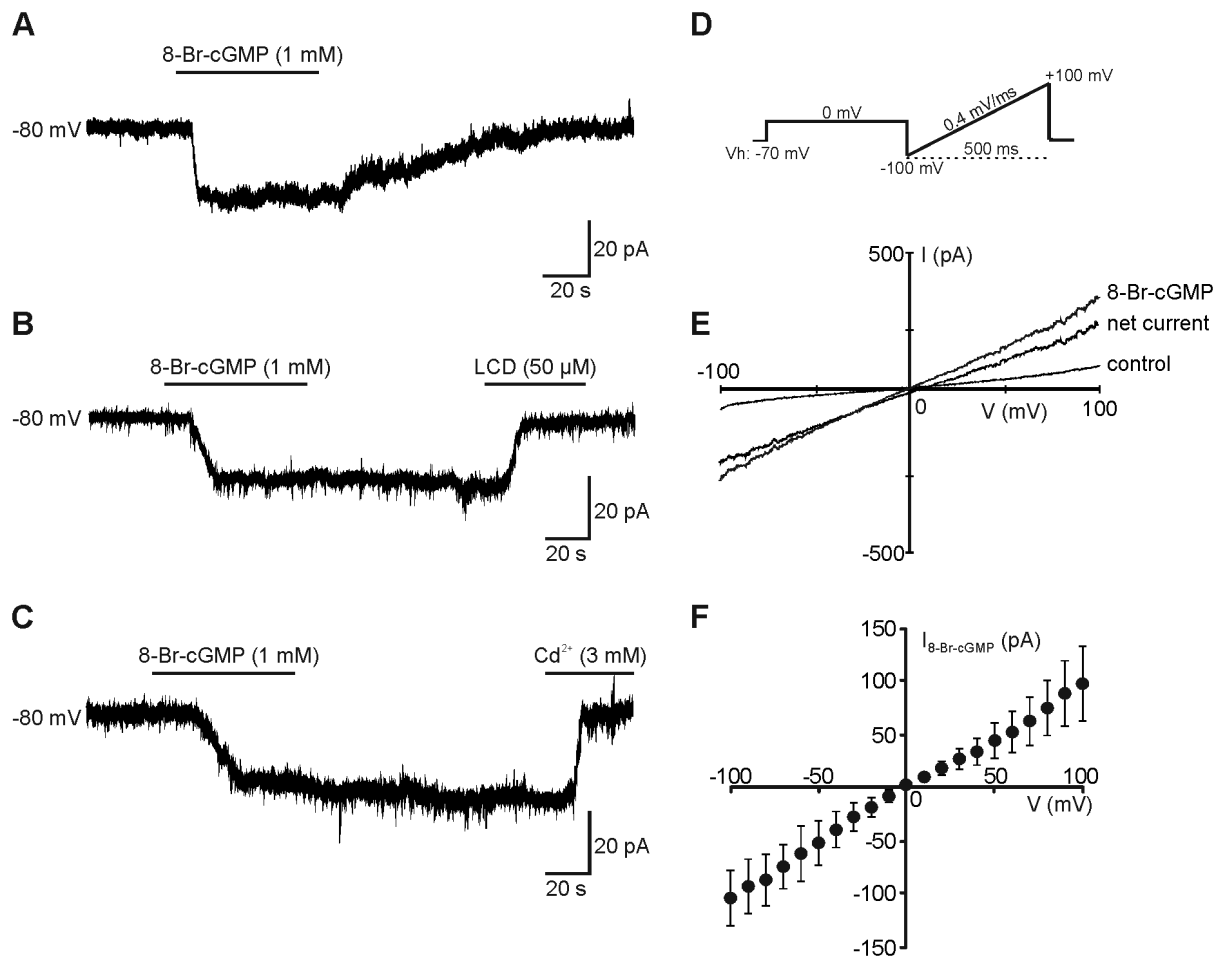
CNG channel-like conductance in differentiating hippocampal NSCs. Representative whole-cell patch-clamp recordings from NSCs grown for three days in differentiation medium and exhibiting morphological features of neurons. Application of 8-Br-cGMP (1 mM) elicited inward currents at holding potential of -80 mV (**A**) which were inhibited by the CNG channel blockers, 50 µM LCD (**B**) and 3 mM Cd^2+^ (**C**). (**D**) Voltage ramp protocol used to evoke leakage currents. (**E**) Typical current traces under control conditions and in the presence of 1 mM 8-Br-cGMP. 8-Br-cGMP-activated current (net current) was obtained by subtraction. (**F**) I–V plot of mean 8-Br-cGMP net currents (*n*=10).

Since CNG channels are voltage-independent non-selective cation channels, we expected that application of the cGMP membrane permeant analogue, 8-Br-cGMP, would elicit non-inactivating inward currents in cells voltage-clamped at negative membrane potentials. Accordingly, cells locally perfused with 8-Br-cGMP (1 mM, for 1-2 min) displayed tonic currents of -22.8±0.9 pA at holding potential of -80 mV (*n*=22 out of 27 cells tested; Figure 4A). The responses were washed out when the cGMP analogue perfusion was replaced by control Tyrode’s solution. These responses were independent on PKG activation because they were observed in the presence of the selective PKG inhibitor, KT5823 (1 µM), which was dialyzed into the recorded cells *via* the patch pipette solution. No significant changes in membrane currents were, instead, observed when 8-Br-cGMP was applied to cells pre-treated for 3-5 min with the CNG channel blocker LCD (50 µM; -1.2±0.3 pA, *n*=12). Furthermore, when LCD was applied once the response to 8-Br-cGMP had fully developed it caused a marked reduction of current amplitudes (-98.4±5.2%; *n*=6; *P*<0.05; Figure 4B). 8-Br-cGMP-induced currents were also virtually abolished by the divalent cation, cadmium (3 mM; -99.3±0.4%; *n*=6; *P*<0.05; Figure 4C), which is a typical feature of CNG-channel mediated responses [42,43,53].

In undifferentiated NSCs (D0) held at -80 mV application 8-Br-cGMP did not elicit currents of significant amplitude (-0.6±0.2 pA; *n*=12).

To better investigate the nature of the 8-Br-cGMP-elicited currents in differentiating NSCs we used a voltage-ramp protocol, described in a previous paper [42], which generated a continuous current-to-voltage (I–V) plot. Leakage currents were recorded at holding potential of -70 mV and subsequently evoked by ramping membrane voltage from -100 mV to +100 mV at a rate of 0.4 mV/ms in the absence and in the presence of 8-Br-cGMP. The voltage-ramp stimulation protocol was preceded by a depolarizing step from -70 mV to 0 for 500 ms to prevent contamination by voltage-dependent inactivating currents, including those flowing through non-L voltage-gated Ca^2+^ channels [49] (Figure 4D). Subtraction of the current recorded under control conditions from that recorded in the presence of 8Br-cGMP revealed a net ramp current at both negative and positive potential with a mean reversal potential of about 0 mV, which is compatible with the opening of non-selective cation channels (*n*=10; Figure 4E,F). Taken together these results indicate that CNG channels are functional in NSCs differentiating to neurons.

### The blockade of CNG channels does not affect NSC proliferation but decreases their neuronal differentiation

We then ascertained whether CNG channels channels, potentially activated by endogenous cyclic nucleotides, played a functional role in NSC proliferation and/or in their differentiation toward the neuronal phenotype. To test our hypothesis we studied NSC proliferation and neuronal differentiation in the presence of the CNG channel blocker LCD [42,43,54].

**Figure 5:**
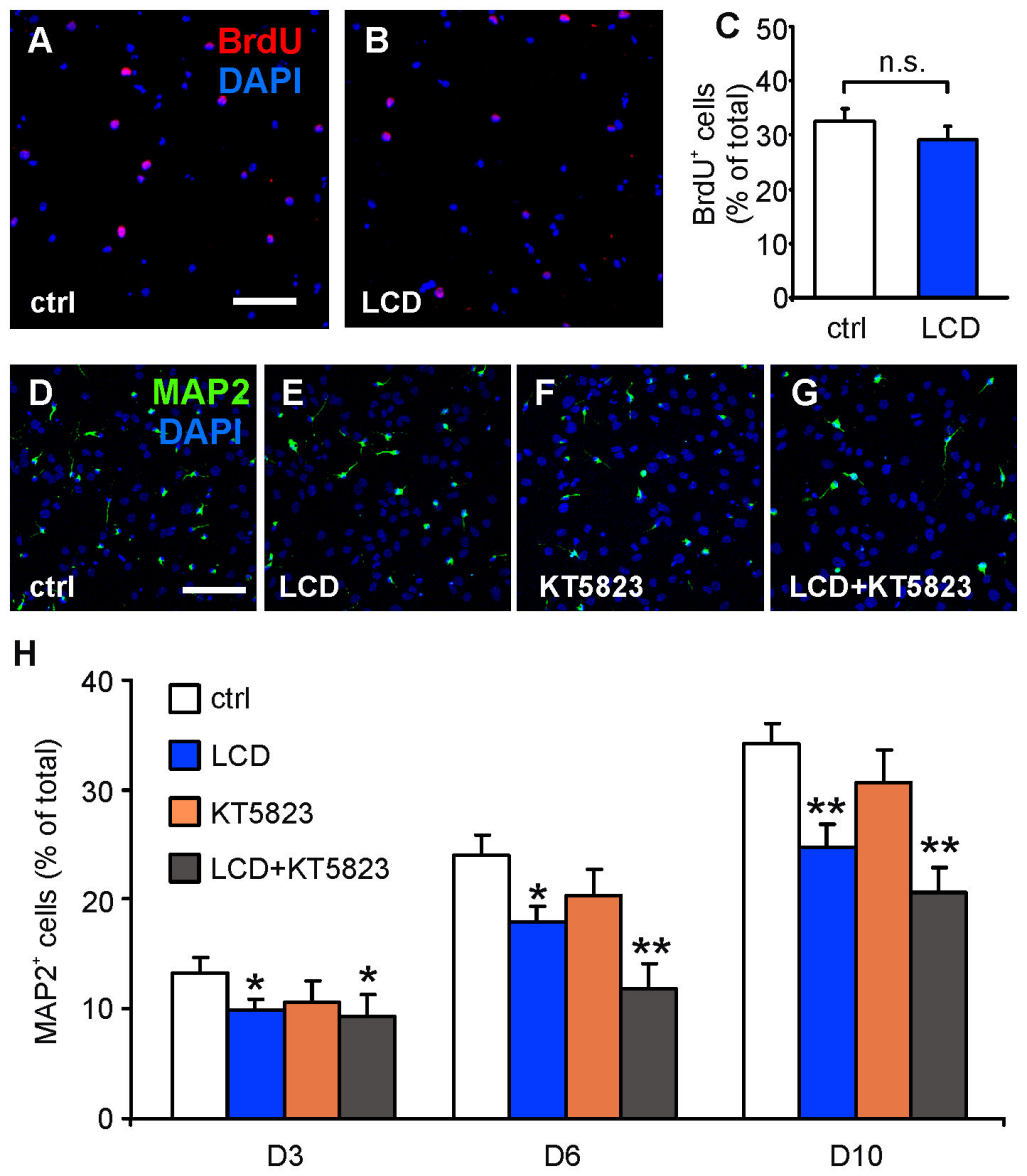
Blockade of CNG channels reduces neuronal differentiation of hippocampal NSCs but does not affect their proliferation. (**A**,**B**) Representative images of BrdU-positive cells (red) in NSC cultures grown in (**A**) normal proliferation medium and (**B**) in the presence of the CNG channel blocker, LCD (50 µM). (**C**) Bar graph showing the percentages of cells incorporating the proliferation marker BrdU in control and LCD-exposed NSC cultures. (**D**–**G**) Representative images of MAP2^+^ NSCs at D6 in control differentiative medium (**D**) and after 3 day-exposure (D1-D3) to 50 µM LCD (**E**), 1 µM KT5823 (**F**) and LCD plus KT5823 (**G**). Cell nuclei (DAPI^+^) are labeled in blue. Scale bars: 75 µm. (**H**) Bar graph showing the percentages of NSCs differentiating towards the neuronal phenotype (MAP2^+^) at D3, D6 and D10 in control conditions and in the culturing conditions described in the graph legend. Error bars show SEM values. Statistical significance was assessed by ANOVA (F_3,52_=3.80, *P*<0.05, at D3; F_3,74_=7.45, *P*<0.0005, at D6; F_3,84_=11.6, *P*<0.0001, at D10). The Dunnett’s post-hoc test was used for multiple comparisons: *P< 0.05 and **P<0.001 vs. control; n.s., not significant *P*-value.

Undifferentiated dividing NSCs (D0) were identified by immunocytochemical detection of BrdU incorporation. Cells were exposed to BrdU (5 µM for 4 h) after 24 h culture in proliferation medium in the absence (control condition) and in the presence of LCD (50 µM). Immunoreactivity for the proliferation marker was observed in 32.6±2.2% of the control cells (529/1568; Figure 5A,C) and it was not significantly different from that observed in LCD-treated cells (29.2±2.4% [506/1608]; Figure 5B,C), thus suggesting that CNG channels, although expressed in undifferentiated NSCs, do not affect their proliferation.

To investigate the role of CNG channels in neuronal differentiation of hippocampal NSCs, the percentage of MAP2^+^ cells was quantified by normalizing the number of MAP2^+^ cells to the total number of cell nuclei labeled with DAPI on D3, D6 and D10 cultures that had been exposed or not to LCD. LCD (50 µM) was added to the differentiation medium at D1 and left for the following two days. The percentage of MAP2^+^ cells from cultures that had been incubated with LCD was significantly lower (9.9±1.1% [254/2566]) compared to control NSCs at D3 (13.3±1.4%, *P*<0.05; Figure 5H). The decrease in neuronal yield (about 25-30%) was similar at D6 and D10 (Figure 4H). In particular, the percentages of MAP2^+^ cells were 18.0±1.3% (601/3137) in LCD vs. 24.0±1.9% in control NSCs at D6 (*P*<0.05; Figure 5D,E,H) and 24.7±2.2% (674/2898) in LCD vs. 34.2±1.9% at D10 (*P*<0.001; Figure 5H).

We also continuously exposed NSCs to 50 µM LCD for 6 days and, under these experimental conditions, neuronal yield was not significantly different from that obtained when LCD was added to the differentiation medium for 3 days (17.7±2.2% vs. 18.0±1.3%, respectively).

The decrease of MAP2^+^ cells in LCD treated-cultures was associated with an increase of GFAP^+^ cells and a decrease of nestin^+^ cells. In LCD-treated cultures at D6, the percentage of GFAP^+^ cells was raised from 72.9±3.5% to 81.2±3.2% (*P*<0.05) and the percentage of nestin^+^ cells was reduced from 5.5±0.9% to 2.5±0.5% (*P*<0.05).

Collectively, these data suggest that CNG channels are activated by endogenous levels of cyclic nucleotides in hippocampal NSCs and promote their neuronal differentiation.

We also tested the possible effects of endogenous cGMP on its most common target in the CNS, i.e., the PKG, and the possible interaction between CNG- and PKG-mediated pathways. As shown in Figure 5H, incubation with the PKG blocker, KT5823 (1 µM, same treatment protocol used for LCD), slightly, but not significantly, reduced NSC neuronal differentiation: the MAP2^+^ cells were 10.6±2.0% at D3 (148/1550), 20.3±2.4% at D6 (384/1800), and 30.7±3.0% at D10 (574/1957). When NSCs were incubated with both LCD and KT5823 the inhibitory effects of the two blockers on neuronal differentiation were additive and highly significant (*P*<0.05 at D3; *P*<0.0001 at D6; *P*<0.0001 at D10 vs. control; Figure 5H). These data suggest that cGMP affects the neuronal differentiation of NSCs through independent intracellular pathways involving both CNG channels and PKG.

### CNG channel blockade prevents the increase of NSC neuronal differentiation induced by exogenous cGMP

**Figure 6:**
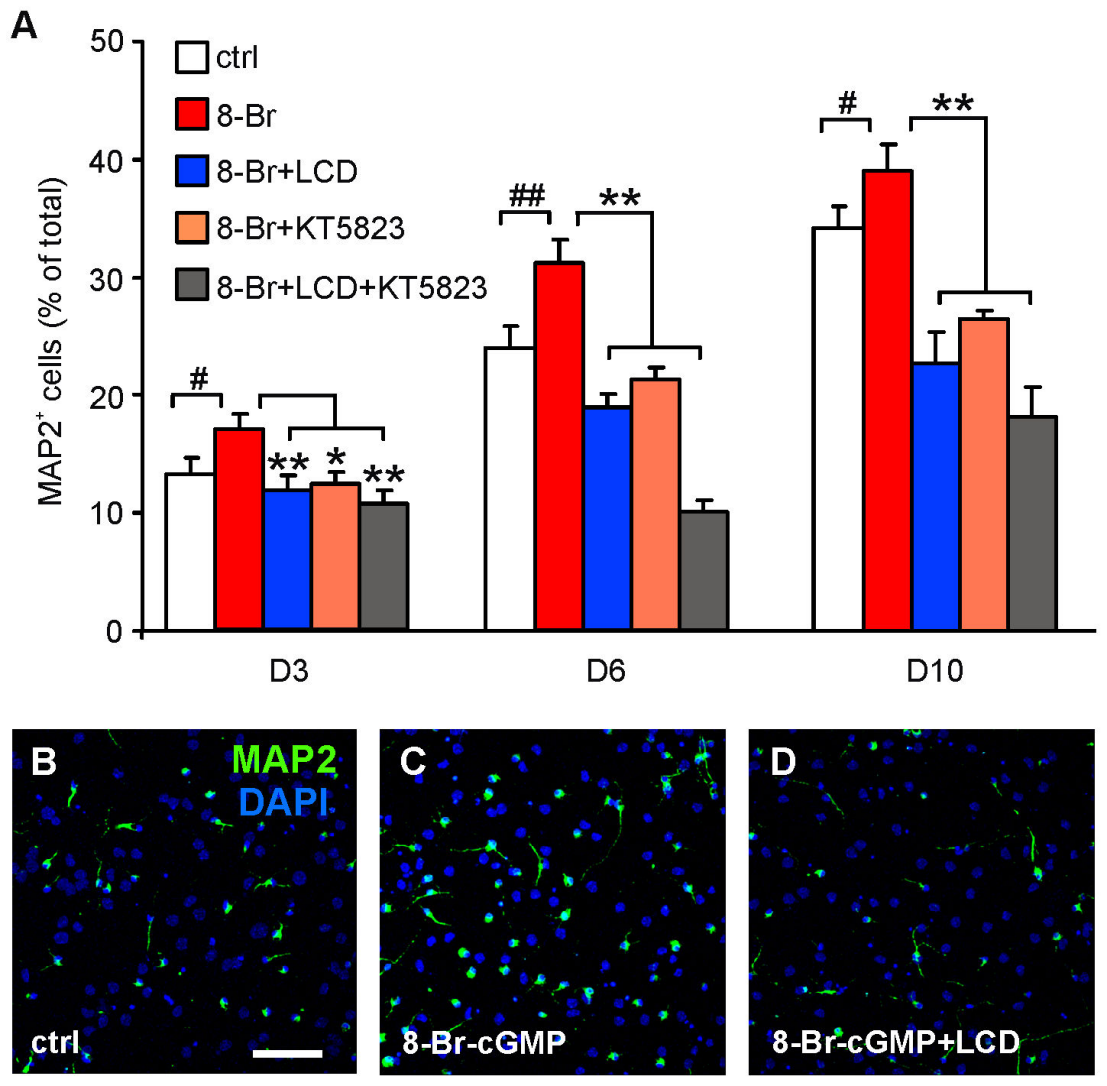
The cGMP analogue, 8-Br-cGMP, promotes NSC neuronal differentiation through CNG channel activation. (**A**) Summary bar graph showing an increased number of MAP2^+^ cells compared to controls following treatment with 8-Br-cGMP (8-Br). This effect was abolished by CNG channel and PKG blockers at D3, D6 and D10. Statistical significance was assessed by ANOVA (F_3,76_=8.25, *P*<0.0001, at D3; F_3,85_=38.5, *P*<0.0001, at D6; F_3,87_=32.8, *P*<0.0001, at D10). The Dunnett’s post-hoc test was used for multiple comparisons: #*P*<0.05 and # #*P*<0.001 vs. control; *P<0.05 and **P<0.001 vs. 8-Br-cGMP-treated NSCs. (**B**–**D**) Representative images of MAP2^+^ NSCs at D6 in control differentiation medium (**B**) and after exposure to 1 mM 8-Br-cGMP (**C**) or 8-Br-cGMP plus 50 µM LCD (**D**). Scale bar: 75 µm.

To confirm that CNG channels are the target of cGMP action in hippocampal NSCs we increased the levels of this cyclic nucleotide by applying its membrane permeant analogue, 8-Br-cGMP, and evaluated NSC proliferation and differentiation in the presence and in the absence of blockers of CNG channels and PKG. In our experimental conditions 8-Br-cGMP (1 mM) failed to affect the rate of NSC proliferation as no significant changes occurred in the percentages of BrdU^+^ cells in 8-Br-cGMP-treated cultures compared to control NSCs (34.5±2.4% [529/1568] vs. 32.6±2.2% in controls). These data confirm that cGMP signaling does not play a role in hippocampal NSC proliferation as suggested by the lack of LCD’s effects we observed in the previous set of experiments. Conversely, the experiments on differentiating NSCs confirmed that cGMP positively modulates NSC differentiation toward neuronal phenotype and its effects are, at least in part, mediated by CNG channels. Indeed, in NSCs that had been exposed to 8-Br-cGMP (1 mM) for three days (D1-D3) the percentage of MAP2^+^ was increased compared to controls (17.2±1.3% [411/2399] vs. 13.3± 1.4% in control NSCs; *P*<0.05; Figure 6A). Increased percentages of MAP2^+^ cells were also observed between 8-Br-cGMP-treated and control cells at D6 (31.3±1.3% [1363/4310] vs. 24.0±1.9%; *P*<0.001; Figure 6A,C) and D10 (39.1±2.0% [1532/3746] vs. 34.2±1.9%; *P*<0.05; Figure 6A). At D6 we also found that 8-Br-cGMP treatment reduced the percentage of GFAP^+^ cells from 72.9±3.5% to 63.3±4.9% (*P*<0.05) without changing the percentage of undifferentiated nestin^+^ cells (5.4±1.5% in 8-Br-cGMP-treated cells *vs.* 5.5±0.9% in controls).

As shown in Figure 6A, LCD treatment completely abolished the effects of 8-Br-cGMP at all time-points studied: MAP2^+^ cells were 11.9±1.2% (308/2515) at D3 (*P*<0.001 vs. 8-Br-cGMP-treated NSCs); 18.9±1.2% (505/2657) at D6 (*P*<0.0001); and 22.7±2.7% (486/2160) at D10 (*P*<0.0001). These results support our conclusion that CNG channels are the target of cGMP in NSCs and they have a role in their neuronal differentiation. It is of interest to note that LCD treatment reduced the proportion of MAP2^+^ cells below the control levels supporting our hypothesis of CNG channel tonic activation by endogenous cyclic nucleotides.

Furthermore, application of the PKG blocker KT5823 (1 µM) exerted a significant inhibitory effect on neuronal differentiation, the percentages of MAP2^+^ cells being 12.4±0.6% (364/2880; *P*<0.01 vs. 8-Br-cGMP-treated NSCs) at D3, 21.4 ± 1.4% (539/2521; *P*<0.0001) at D6 and 26.5±1.9% (768/2960; *P*<0.0001) at D10 (Figure 6A). The co-application of the two blockers produced a stronger effect on NSC neuronal differentiation (10.8±1.1% at D3 [176/1900], *P*<0.001 vs. 8-Br-cGMP-treated NSCs; 10.1±1.0% [260/2096] at D6, *P*<0.0001; 18.3±2.5% [384/2128] at D10, *P*<0.0001).

## Discussion

Our study provides novel evidence supporting a role for CNG channels in adult hippocampal neurogenesis. Immunofluorescence and Western blot experiments revealed that hippocampal NSCs *in vitro* and *in situ* express CNG A subunits of rod (CNGA1) and olfactory-type (CNGA2) and that the channel expression persists in immature and mature DG granule neurons. Additionally, in vitro experiments indicate that activation of CNG channels contribute to NSC differentiation toward the neuronal phenotype without affecting the proliferation of undifferentiated cells. The functional role of CNG channels in NSCs is supported by electrophysiological data showing CNG-channel-like conductances in NSCs differentiating toward neuronal phenotype.

Considerable evidence now exists that CNG channels represent a novel major target for cyclic nucleotide action in the CNS. Functional expression of CNG channels, especially of rod and olfactory types, has been demonstrated in neurons and astrocytes of the rodent brain [33,38,41,42,50–52,55–57]. In neurons cation influx through CNG channels mediates membrane depolarizations [41,58,59]. Moreover, it has been established that Ca^2+^ influx through activated CNG channels enhances neurotransmitter release and influences synaptic plasticity [36,37,39,40] and it has been hypothesized to play a fundamental role in the CNG-mediated regulation of glial function [42].

Interestingly enough, previous studies have also suggested a role of CNG channels in CNS development. In rats, both molecular and electrophysiological experiments have detected CNG channel expression in immature hippocampal neurons before synapse formation [60,61], suggesting that CNG channels could function at early stages of neural development. In the visual cortex the rod and olfactory subtypes are developmentally regulated, being present at the time of migration and rapid dendritic outgrowth [55]. In this context several experimental findings also support a specific role of CNG channels as guidance molecules, in growth cone formation and synaptic bouton maturation [38,43,52].

Here we provide novel evidence that CNG channels are expressed in primary cultured hippocampal NSCs obtained from postnatal mouse brain. NSCs were identified by immunocitochemical detection of nestin, which is a marker of neural stem/progenitor cells, as well as by their ability to proliferate, as shown by BrdU incorporation. Consistent with our hypothesis of a role for CNG channels in adult neurogenesis their expression was also confirmed in the neurogenic niche of the adult mouse hippocampus where nestin positive cells displayed both CNGA1 and CNGA2 immunoreactivities. Our *in vitro* and *in situ* data also show that expression of CNG channels persists in immature and more mature neurons. This observation is in agreement with previous studies demonstrating the expression of CNGA1 and CNGA2 at mRNA and protein levels in neurons from different hippocampal subfields including the DG granule cells [51,52].

Several data support a role of CNG channels in hippocampal neurons: first, the existence of cGMP-gated conductances and also of Ca^2+^ transients has been demonstrated in embryonic hippocampal neurons and attributed to olfactory-type CNG channel activation [52,61]. By using CNGA3 knockout mice Michalakis and co-workers [39] demonstrated that these channels are critically involved in the regulation of synaptic plasticity in the hippocampus. Here we provided evidence of putative CNG channel conductances in NSCs at early stage of neuronal differentiation (D3) which support their role in the process of neurogenesis. Currents we recorded were similar, though much smaller, to cGMP-gated-conductances previously described in cultured embryonic rat hippocampal neurons [52,61], as well as to those reportedly associated to CNG channel activation in other cell types in terms of time course, reversibility and pharmacological sensitivity to CNG channel blockers [38,41,42,53,60].

In the hippocampal neurogenic niche, CNG channels are good candidates for transducing signals received by adult NSCs influencing the complex process of neurogenesis. These channels are activated by low micromolar concentration of cyclic nucleotides, they are voltage-independent and do not inactivate, allowing ion influx as long as cyclic nucleotide levels are elevated. Furthermore, CNG channels and especially CNGA2-type are highly permeable to calcium, which play a fundamental role in promoting NSC differentiation toward neuronal phenotype [17,45,62].

We found that treatment of hippocampal NSCs with the CNG channel blocker LCD during the first three days in differentiating conditions resulted in a decrease of neuronal yield by about 25%, which was maintained in the following 7 days of culture. These data indicate that CNG channels are activated by basal level of cyclic nucleotides in NSCs and participate in their neuronal differentiation.

CNG channels, especially those bearing the CNGA2 subunit, are sensitive to cGMP and cAMP, both of which have been involved in signaling pathways modulating adult neurogenesis. In particular, it has been well demonstrated that cAMP plays an important role in the differentiation and maturation of newborn neurons in the hippocampus. Chronic treatment with rolipram, which inhibits the selective cAMP degrading phosphodiesterase, PDE4, increases proliferation and survival of newborn neurons in DG [63,64] and PDE-4 KO mice showed increased hippocampal neurogenesis [65]. All these studies linked the effect of cAMP to activation of CREB cascade. Although we cannot exclude that CNG channels may be other targets of cAMP action in NSCs, we focused our study on cGMP signaling which was less characterized.

Data from the literature indicate that increased levels of cGMP, in an animal model of stroke, induced proliferation of progenitor cells in the SVZ and DG and increased the number of immature neurons [25,26,31]. The proliferative effects of cGMP in SVZ NSCs have been attributed to activation of PKG-dependent pathways [66]. A recent study also suggested a role of cGMP during brain development *in vivo* by demonstrating that rats in which cGMP levels had been reduced during embryonic development showed reduced differentiation of stem cells to neurons in the cortex and in the DG of the hippocampus [21].

With regard to adult hippocampal neurogenesis the specific roles of cGMP and CNG channels had not been studied previously. Our data demonstrate that neither blockade of CNG channels nor exposure to the membrane permeant analogue of cGMP, 8-Br-cGMP, affected NSC proliferation. Given the molecular evidence that the channels are expressed at this stage, it is possible that the signaling pathway downstream CNG channel activation is not relevant for NSC proliferation. On the other hand, since our electrophysiological recordings showed the lack of responses to 8-Br-cGMP in proliferating NSCs, it likely that the channels are not functional at this stage. Indeed, several factors affecting CNG-channel functional state and cyclic nucleotide-sensitivity have been reported, including their phosphorylation state, divalent cations, Ca^2+^/calmodulin, diacylglycerol, phospholipids and, more recently, insulin receptor activation [67–70]. Further studies are needed to elucidate the mechanisms of CNG channel modulation in NSCs at different stages of development.

When 8-Br-cGMP was applied to differentiating NSCs (D1 to D3) the percentage of cells labeled with the neuronal marker MAP2 increased compared to control cells at D3. The increased number of MAP2^+^ cells observed at D6 and D10 indicates that enhancement of neuronal differentiation by cGMP in the first stages results in an effective yield of mature neurons. In the presence of the CNG channel blocker, LCD, the effects of 8-Br-cGMP were completely abolished and the neuronal yields at D3, D6 and D10 were even lower compared to untreated cultures. The latter observation is probably related to an additional blockade by LCD of CNG channels activated by intracellular cyclic nucleotides. Accordingly, we found a reduction in the number of MAP2^+^ cells by LCD alone.

Remarkably, our data suggest that CNG channels play a role in fate determination of hippocampal NSCs by promoting their differentiation towards neuronal vs. glial phenotype. Indeed, the increases in MAP2^+^ cells induced by CNG channel activation was accompanied by a decrease in the number of cells acquiring the glial phenotype (GFAP^+^ cells), whereas the blockade of CNG channels increased the number of GFAP^+^ cells. These results are in line with the recently proposed role of NO/cGMP in promoting neuronal differentiation of NSCs during development [21] and point to CNG channels as possible candidates for mediating such effects.

As for the possible involvement of PKG in mediating cGMP effect on NSC differentiation, our data suggested that also this protein kinase plays a role. Indeed, when the PKG blocker, KT5823 was applied alone, it slightly reduced NSC neuronal differentiation and abolished the effect of 8-Br-cGMP. Having not blocked endogenous production of cGMP in our experiments we are not able to define the specific contribution of the two targets in mediating 8-Br-cGMP effects. Co-application of PKG and CNG channel blockers caused poor neuronal differentiation at D3, D6 and D10, thus indicating that cGMP signaling plays an important role in this process. The effects of the two blockers on neuronal differentiation were higher than those of each drug, indicating that PKG and CNG channels affect NSC differentiation by acting through independent pathways.

As for the intracellular mechanisms linking the CNG channel activation to enhancement of NSC neuronal differentiation it is plausible to hypothesize that increased Ca^2+^ levels, likely occurring following CNG-channel activation, are involved. Possible pathways downstream Ca^2+^ influx through activated CNG channels may include activation of Ca^2+^-dependent kinases (e.g., Ca^2+^/calmodulin and extracellular signal-regulated, ERK, kinases) resulting in phosphorylation of cyclic AMP responsive element binding protein (CREB) and subsequent upregulation of pro-neuronal genes such as Mash1, Neurogenin1 and NeuroD1, which have all been reportedly implicated in NSC neuronal differentiation [17,45,62,71,72].

Among the possible signals promoting cyclic nucleotide elevation that might activate CNG channels in hippocampal NSCs *in vivo*, NO is of particular relevance. This gaseous molecule represents the major modulator of cGMP levels in the brain [73] and, interestingly, it has been shown to influence adult neurogenesis both in basal conditions and following injury. It should be highlighted that contradictory results have been reported on the effects of NO on neurogenesis. NO was described as a physiological inhibitor of neurogenesis [74,75] whereas other studies demonstrated that NO is a positive modulator of NSC proliferation in the neurogenic niches following stroke or seizures [28,76] as well as during brain development [21]. Different results have been hypothesized to be related to different NO concentrations [28] or to cellular (neurons or glia vs. NSCs) and subcellular (cytoplasmatic vs. nuclear) localization of NO synthetizing enzymes [30]. Although the mechanisms underlying NO effects on neurogenesis have not been fully explored, some of its inhibitory effects have been linked to the activation of cGMP-independent pathways such as protein S-nitrosylation [74], whereas enhancement of neurogenesis by NO *in vivo* has been clearly related to cGMP at least by one study [21]. Based on our results it is tempting to speculate that NO positive modulation of adult neurogenesis may be mediated by cGMP and involve CNG channel activation, whereas NO’s inhibitory effects are mainly linked to its cGMP-independent effects.

Besides NO, other possible candidates for promoting CNG channel activation in hippocampal NSCs include natriuretic peptides, which increase cGMP levels through activation of particulate guanylate cyclases acting as natriuretic peptide receptors in several cell types including NSCs [77–79].

In conclusion, our findings provide novel evidence that hippocampal NSCs express CNG channels and their activation by cGMP promotes neuronal differentiation. These results, besides highlighting the relevance of cGMP signaling pathway in adult neurogenesis, reveal an additional role of CNG channels in the CNS in the regulation of an important aspect of brain physiology. This knowledge takes on particular relevance considering that evidences support a link between the role of cGMP signaling in hippocampal neurogenesis and the pathophysiology of major depressive disorders as well as antidepressant treatment responses [80]. Additionally, understanding how cGMP and its downstream targets contribute to adult neurogenesis may help to design well-grounded therapies for stimulating endogenous neurogenesis for neural repair and to treat or delay neurodegenerative diseases where this process is impaired.
